# Sri Lankans vigilant after bidding farewell to malaria

**DOI:** 10.2471/BLT.17.020317

**Published:** 2017-03-01

## Abstract

Sri Lanka was certified malaria free last year. The challenge now is to maintain that status. Sophie Cousins reports.

Between 1934 and 1935, one-quarter of Sri Lanka’s population contracted malaria. Over the course of seven months, 80 000 people lost their lives.

It was the worst malaria outbreak recorded and a dark chapter in the island’s history.

“My grandparents used to talk about the 1935 malaria outbreak and how devastating it was,” says Dr Hemantha Herath, director of the Anti Malaria Campaign – which is run by the Ministry of Health – in his office in Colombo, the capital.

“There was no one to bury the dead. Everyone was either sick with malaria or had died of it.”

“There was no one to bury the dead. Everyone was either sick with malaria or had died of it.”

Hemantha Herath

Last September, Sri Lanka was certified malaria free by the World Health Organization (WHO), becoming the second country in WHO’s South-East Asian Region after the Maldives, another island state, to achieve this.

To become officially malaria free, a country must report zero indigenous cases for three consecutive years. Sri Lanka’s last case was reported in October 2012.

The road to elimination was long and difficult in this country of 20.7 million people, where malaria was endemic in 21 of its 25 districts.

Following the 1935 outbreak, major elimination activities were rolled out including the introduction of indoor residual spraying. By 1963, only 17 cases were reported. Malaria elimination seemed within reach and malaria funds were reallocated, drastically reducing surveillance for the parasitic disease.

“We thought we were on the verge of elimination,” says Dr Janakan Navaratnasingam, a medical officer for communicable diseases at the WHO Country Office in Sri Lanka.

“But vector control measures were not followed properly and antimalarial staff were moved to other control programmes.”

These factors led to a major outbreak with an estimated 500 000 cases in the late 1960s.

Antimalarial activities were scaled up again, as part of a national control programme, but many cases were reported throughout the 1970s and 1980s. In the 1990s, the Anti Malaria Campaign shifted its focus from vector to parasite control by active and passive case detection; referring to this change in strategy, Herath says: “It was an important policy decision”.

“We tried to detect as many sick people as possible and treat them to substantially reduce their parasite load,” Herath recalls.

The campaign also increased coverage and use of bed nets. Mobile malaria clinics were deployed in high transmission areas allowing for quick and effective treatment to reduce the parasite reservoir and slow transmission.

In addition, health education was provided and insecticide-treated bed nets were introduced, leading to a huge reduction in cases from 2000 onwards.

The first symptoms of malaria – fever, headache, chills and vomiting – are mild and often difficult to recognize as malaria. If not treated promptly, malaria can progress to severe illness and, sometimes, death, particularly in children who have less immunity.

A major challenge for malaria control in Sri Lanka was the armed conflict that raged from 1983 to 2009 in the north and east of the island. Fortunately, the conflict parties supported the malaria prevention and control efforts throughout the war, according to Janakan.

“When health workers went to the rebel-held areas to carry out antimalarial activities, the rebels never opposed them because they were also suffering from malaria,” Janakan says.

Experts attribute Sri Lanka’s success in beating malaria to several factors including its education system, with high literacy rates of nearly 93% in 2015, its good road infrastructure and its health system.

“Sri Lanka started building its public health infrastructure in the 1920s. Today most people can access health care within a few kilometres of their homes,” says Dr Jacob Kumaresan, WHO Representative to Sri Lanka.

For Dr Kamini Mendis, who worked as a malaria researcher in the capital Colombo throughout the 1980s and 1990s, Sri Lanka’s success is also a result of training malaria control programme officers.

“Instead of just blindly putting up bed nets, they took a more evidence-based approach to malaria control. Malaria is a disease that is very focal and local and needs to be attacked in a specific way, not by taking a blanket approach,” says Mendis, who worked with the WHO malaria programme in Geneva. 

But while the country’s achievement is a public health success story, malaria could return. “We have no more malaria cases, but that does not mean the mosquito is no more. There are plenty of mosquitoes. The receptivity is always there, so the country is always vulnerable,” says Janakan.

“We have no more malaria cases, but that does not mean the mosquito is no more.”

Janakan Navaratnasingam

Public health authorities are working to prevent malaria returning by identifying and managing imported cases, and by ensuring that funding for such surveillance is maintained.

These efforts target Sri Lankans who visit malaria endemic areas and come home, such as business people, tourists, pilgrims and United Nations peacekeeping forces, and people from endemic countries who visit Sri Lanka.

Malaria is transmitted to humans by the bite of an infected female *Anopheles* mosquito. It is caused by five protozoan species of the genus *Plasmodium, *two of which are found in Sri Lanka: *P. falciparum* and *P. vivax, *Mendis explains. 

When people with *P. vivax* are not treated properly, the parasites remain in their liver and can lead to renewed bouts of sickness. Incomplete treatment is sometimes the case in India, Sri Lanka’s neighbour to the north, which has a high incidence of malaria and limited access to treatment.

“Our biggest danger is our proximity to India,” says Mendis, explaining that Sri Lanka records at least one imported case a week, mainly from India. Doctors often assume that these patients have dengue fever and there is concern in the public health community in Sri Lanka that malaria could make a comeback.

In response to the risk of imported cases, the health ministry’s Anti Malaria Campaign is running programmes to raise more awareness of the threat.

“We’re trying to educate doctors. We tell them that when they come across patients with fever, they should ask for the person’s travel history and test them for malaria,” Herath says.

He adds that the campaign also provides up to six months’ prophylaxis (preventive) treatment to Sri Lankans going to malaria-endemic areas, and services for the screening every three months of Sri Lankans who have returned from endemic areas.

For Mendis, the only solution to prevent the re-introduction of malaria is effective surveillance, rapid diagnosis of malaria and effective treatment.

But to do that, funding must not be reduced, Kumaresan warns.

“When malaria is not there, then they might say: ‘Why should I give you money?’”

“Funding for malaria may be reallocated to other disease programmes. That’s what happened 50 years ago, so we really have to be vigilant that we don’t fall into that trap again.”

For Dr Kolitha Wickramage, who worked in northern Sri Lanka 10 years ago with WHO providing health care to displaced people and other communities affected by the conflict, control measures at the country’s sea ports and airports are essential to prevent re-importation.

“An evidence-based and humane approach to point of entry screening of travellers arriving from malaria-endemic countries is important, as reflected in Sri Lanka's national migration health policy,” he says

“Some population groups are at high risk of re-introducing malaria, such as irregular migrants and peacekeepers,” says Wickramage, who is now the global migration health research and epidemiology coordinator at the International Organization for Migration, the United Nations agency for migration.

In 2014 alone 32 cases of *P. falciparum* were detected in 534 irregular migrants, who returned to Sri Lanka from West Africa after failed attempts to smuggle them into other countries.

In recent years, Anti Malaria Campaign officers have been testing these migrants as well as returning peacekeepers with rapid diagnostic tests when they enter the country. Those who do not test positive on the border are tested again within two weeks of their arrival. Those who test positive receive treatment and follow-up. 

Wickramage calls for a strategy that also examines inbound travel patterns and closely monitors migrants from malaria-endemic zones to better understand the risk.

He suggests involving the tourism industry, travel operators and Sri Lankan embassies all over the world to alert travellers to the risk of malaria re-importation.

“There should be better resources and better thinking around this because there’s probably an underestimation of the total number of imported cases,” he says.

Back at his office, Herath reminisces about the 10 years he spent working as a medical officer with the Anti Malaria Campaign in the north-west of the country in the 1990s, when malaria was rampant.

“At the time, everyone in my family was asking, ‘why are you going to these areas?’ Thank God not me or anyone in my family got malaria,” he says.

**Figure:**
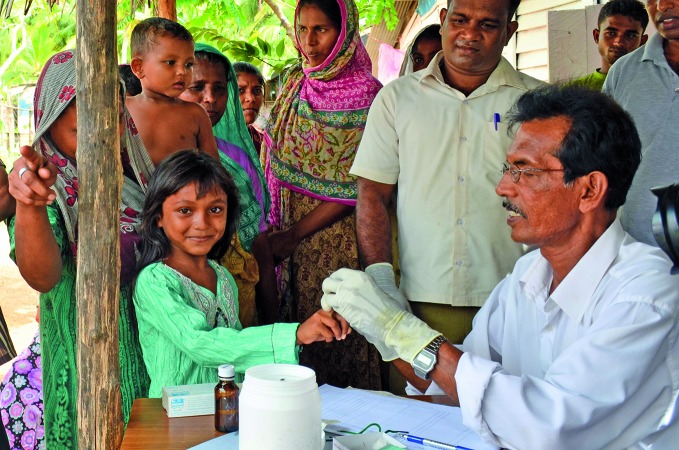
Girl receives a blood test as part of routine malaria surveillance.

Ministry of Health, Sri Lanka

**Figure:**
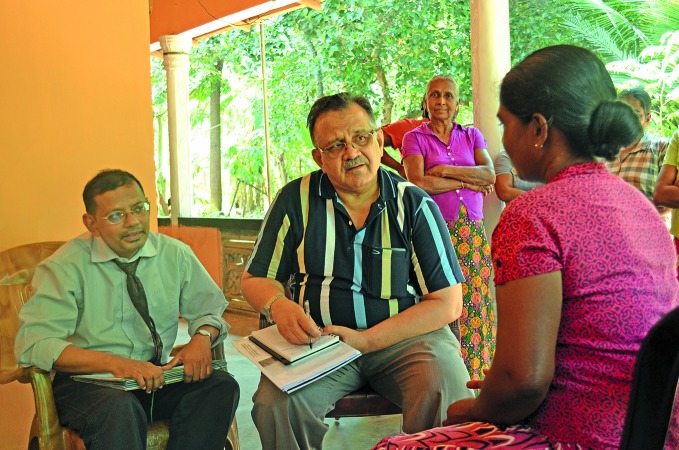
Dr Chandrakant Revankar (right) and Mr S R Jayanetti (left)interview community members.

Ministry of Health, Sri Lanka/Dr Risintha Premaratne

